# Kinesin genes KIF4A, KIF20A and KIF11 as prognostic biomarkers in lung adenocarcinoma by integrative bioinformatic analysis and experimental validation

**DOI:** 10.1038/s41598-025-29206-9

**Published:** 2025-12-29

**Authors:** Huanqin Wang, Lu Zhou, Fengxiang Huang

**Affiliations:** Department of Respiratory Medicine, The First Affiliated Hospital, Zhengzhou University, Zhengzhou, 450052 China

**Keywords:** KIF, LUAD, Prognostic biomarker, Gene signature, Proliferation, Migration, Non-small-cell lung cancer, Protein sequence analyses

## Abstract

lung adenocarcinoma (LUAD) causes a large number of deaths worldwide each year. The pivotal involvement of Kinesin superfamily proteins (KIFs) plays a critical role in the progression of LUAD. This study primarily investigates the biological functionalities associated with specific genes within the KIF family while concurrently developing prognostic signatures of LUAD. The LUAD databases were examined to analyze differentially expressed genes (DEGs) associated with KIFs. Prognostic genes related to these DEG-KIFs were identified through univariate Cox analysis. A risk model incorporating these selected genes was constructed using LASSO regression technique. The predictive capability of this model was evaluated through survival analysis, while the functional role of KIF genes was investigated in vitro. The prognostic significance of a gene signature comprising 3 KIF genes (KIF4A, KIF20A, and KIF11) was confirmed. Overall survival analysis demonstrated the robust performance of the risk model. A nomogram and calibration curves based on 3 hub genes showed excellent prognosis predictive performance. The risk score of this risk model is closely related to the activation of ECM signaling pathway and the infiltration of multiple immune cells. The expression levels of the 3 KIF genes were also closely related to the dosages of various LUAD therapeutic drugs. In vitro experiments revealed that silencing KIF4A, KIF20A, and KIF11 effectively suppressed cell proliferation, invasion, and migration in LUAD cells. We found that this signature (KIF4A, KIF20A, KIF11) could be a potential biomarker and treatment target for LUAD patients. And our study plays a critical role in exploring the clinical application of KIFs for LUAD.

**Supplementary Information:**

The online version contains supplementary material available at 10.1038/s41598-025-29206-9.

## Introduction

Lung cancer is a prevalent global malignancy. The predominant type of primary lung cancer cases (85%) is attributed to non-small cell lung cancer (NSCLC), which includes various subtypes including lung adenocarcinoma (LUAD), lung squamous cell carcinoma (LUSC), bronchioloalveolar carcinoma, and large cell carcinoma^1^. Among these subtypes, LUAD represents the most frequently observed histological subtype within NSCLC^2^. Patients with LUAD are usually diagnosed at an advanced stage. Traditional prognostic techniques such as histopathological assessment and tumor staging systems have shown limited effectiveness^2^. Therefore, it becomes imperative for us to identify specific, reliable, and non-invasive tumor biomarkers that can facilitate the diagnosis and prognosis assessment of LUAD.

It has been proven that disrupting the formation of the mitotic spindle to interfere with cellular processes is an effective approach for treating cancer^3^. Targeted medications (such as paclitaxel and docetaxel microtubules) that act on microtubules (MT) can disrupt MT dynamics, prolong the mitosis stage, ultimately leading to cell death^4^. Kinesin superfamily proteins (*KIFs*) are a type of molecular motors based on MT. Since their initial discovery in 1985, it has been found that the *KIF* superfamily consists of 45 family members^5^. These members possess highly conserved movement domain structures which serve as binding sites for MT movement. *KIFs* can travel along the MT track using unidirectional motion. It has also been demonstrated that *KIFs* played various roles in intracellular transport or cell division, including involvement in organelles, protein complexes, mRNA transport, and participation in chromosome segregation during mitosis and meiosis periods as spindle motors^6^. Numerous studies have indicated abnormal expression of *KIF* superfamily members in different tumors and their involvement in tumor occurrence and development. Some researchers discovered that *KIF17* could facilitate microtubule acetylation to maintain microtubule stability, thereby suppressing invasion and metastasis of breast cancer cells^7^. Liu and his colleagues discovered a noteworthy association between elevated levels of *KIF11* expression and the survival rate of patients with NSCLC^8^. Chen et al. demonstrated that down-regulation of *KIF26B* could inhibit malignant behaviors of NSCLC by blocking *AKT*/*GSK-3β*-mediated *Wnt*/*β-catenin* pathway activation^9^. Currently, clinical trials have utilized several inhibitors that target specific KIF proteins, including AZD4877 which selectively inhibits *KIF11*, and GSK923295 which targets KIFIO^10,11^. Despite advancements in studying individual members of this protein family, the precise role and prognostic significance of *KIFs* in LUAD remain uncertain.

**Fig. 1:**
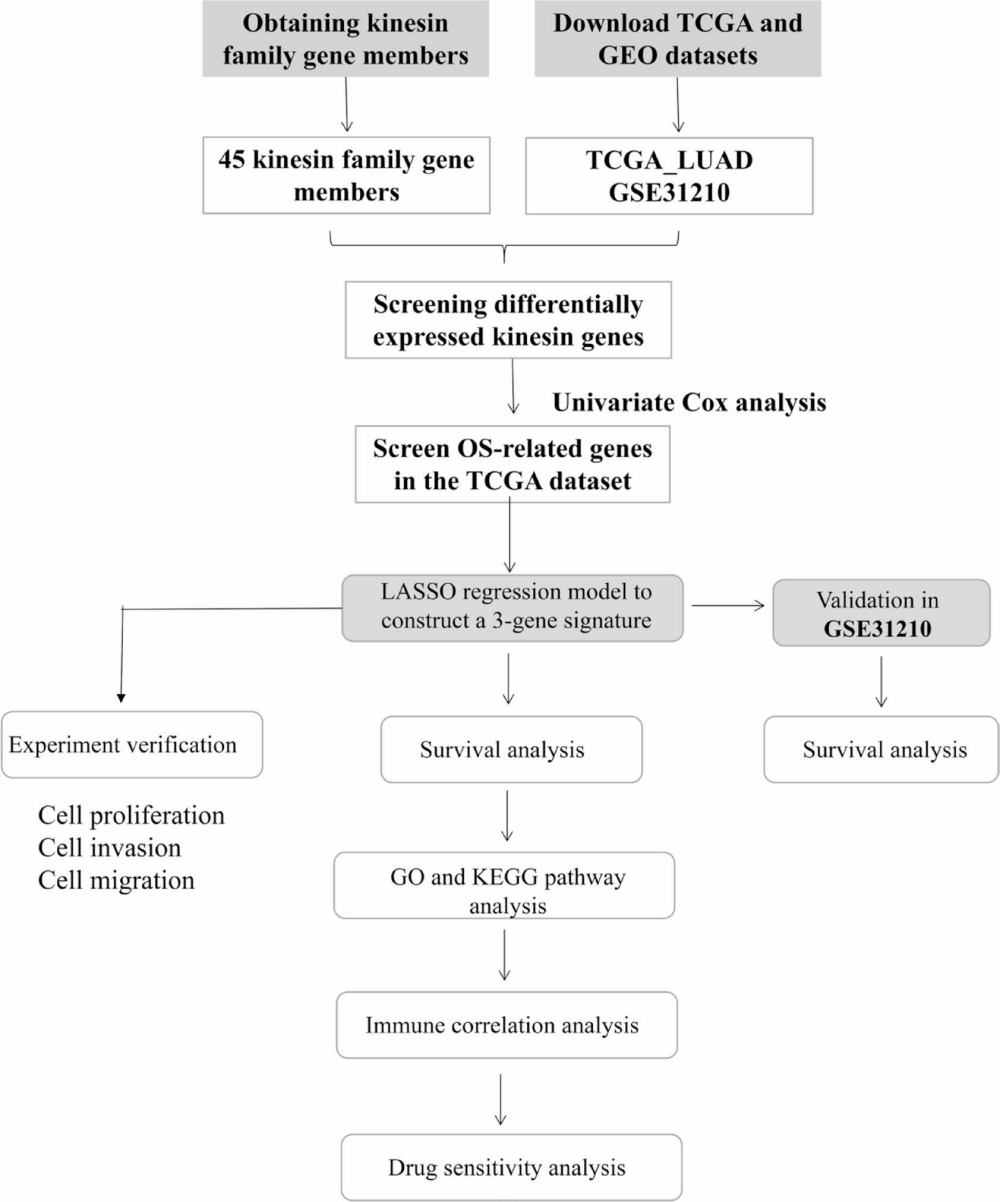
The overall flow diagram of this study.

In this research, we conducted an analysis on the mRNA gene expression of numerous tumor samples from The Cancer Genome Atlas (TCGA) and Gene Expression Omnibus (GEO). Our focus was on identifying a prognostic signature for LUAD by examining 3 *KIFs* that exhibited significant prognostic value. This signature holds promising clinical application potential in predicting prognosis of LUAD patients. The overall methodology employed in this study is visually depicted in Fig. 1.

## Materials and methods

### Data acquisition and processing

This research made use of two databases, specifically TCGA and GEO, to gather information. The Xena Browser at the University of California, Santa Cruz (UCSC) was utilized to access RNA sequencing (RNA-Seq) data and clinical details of LUAD patients, including survival information. Additionally, we sourced the GSE31210 cohort from the GEO database. In the TCGA dataset, we used the LUAD cohort, which includes both tumor and normal tissue samples. The primary tumor samples were categorized as the disease group, while the normal tissue samples served as the control group. A similar approach was applied to the GEO dataset (GSE31210), where samples were divided into tumor and normal tissue groups based on clinical information from the patients. To identify differentially expressed genes (DEGs) between normal and tumor tissues in each LUAD cohort, we employed the LIMMA method available on GEPIA2 (http://gepia2.cancer-pku.cn/#index)^12^. The limma package in R was employed to analyze differential mRNA expression, applying linear models to assess the variation in gene expression across different groups. DEGs were determined based on a cutoff criteria of adjusted *q* value < 0.01 and |log2 fold change (FC)| >1. The DEGs were subsequently visualized using volcano plots. We used Venny 2.1 software to identify common genes between these DEGs and *KIFs*. For external validation purposes, we conducted a validation study using the LUAD cohort GSE31210 from the GEO database.

## Construction and evaluation of DEG-KIF signature

To perform a univariate Cox proportional hazards regression analysis for the identification of prognostic DEG-*KIFs*, we employed the survival R package. This analysis revealed significant associations between certain genes and overall survival (OS) in the TCGA-LUAD dataset. We calculated the Hazard ratios (HRs) with 95% confidence intervals (95% CIs). We considered a significant association with event hazards and a significant impact on survival time when HR > 1 and *P* < 0.05.

The R package “glmnet” was utilized to establish an optimal risk signature from survival-related genes through LASSO penalized Cox regression. The risk score was calculated using the equation: (expression of *KIF4A* × 0.101429576585459) + (expression of *KIF20A* × 0.0892753671059616) + (expression of *KIF11* × 0.0515911609040658). Following that, LUAD individuals were classified into high- and low-risk categories by considering the median risk scores within each group. To examine gene distribution across different groups according to their expression levels in this model, we employed Principal Component Analysis (PCA) with the assistance of the “ggplot2” R package.

To assess the predictive precision of this signature, we performed time-dependent analysis by employing ROC curves and determining Area Under Curve (AUC) values with the assistance of R packages. Specifically, we utilized the “timeROC” R package to perform ROC analysis for 1-year, 3-year, and 5-year periods.

## Enrichment analysis of the DEGs between the low- and high-risk group

The LUAD patients from the TCGA cohort were categorized into two subgroups based on their best risk score. The DEGs between the low-risk and high-risk groups were selected using specific criteria (|log2FC|≥1 and FDR < 0.05). Afterwards, we performed analyses using Gene Ontology (GO) and Kyoto Encyclopedia of Genes and Genomes (KEGG^13–15^ to investigate the DEGs between these two groups. We have taken into account a threshold of adjusted *P* value < 0.05 to identify cellular processes that exhibit statistical significance.

## Tumor microenvironment analysis

We utilized TIMER (http://timer.cistrome.org/) to evaluate the frequency of 6 immune cell infiltrations so as to explore the association between KIF genes and immune infiltration within the TCGA-LUAD dataset^16^.

## Construction and assessment of the nomogram

The significant clinical features and risk scores identified by conducting univariate Cox analysis were used to develop a nomogram with the assistance of R packages ‘survival’ and ‘rms’. The effectiveness of the model was assessed using the concordance index (C-index), whereas calibration plots were employed to evaluate how well predicted survival outcomes aligned with actual observations.

### Kinesin gene expression and drug response

CellMiner is a comprehensive database utilized by the cancer research community. It enables the analysis of more than 100,000 chemical compounds and natural substances across a diverse range of 60 human cancer cell lines (NCI-60)^17^. The findings from CellMiner indicate a correlation between medication sensitivity and the expression of kinesin genes.

## Cells and cell culture

We acquired normal epithelial lung cell line BEAS-2B and two LUAD lines, H1299 and A549 from National Collection of Authenticated Cell Cultures (Shanghai, China). Mycoplasma testing has been done for the cell lines. None of the cell sample was contaminated by mycoplasma. Cell lines were authenticated with an ATCC profile using the short tandem repeat DNA profile method. BEAS-2B and A549 cells were cultured in DMEM medium (Gibco, Carlsbad, CA, USA) supplemented with 10% fetal bovine serum (Gibco), penicillin (100 IU/ml), and streptomycin sulfate (100 µg/ml) (Solarbio, Beijing, China). Similarly, the H1299 cell line was cultivated under identical conditions using RPMI 1640 medium (Gibco) along with the same supplementation.

## Cell transfection

We opted to utilize A549 and H1299 cells for subsequent experiments. All siRNAs targeting *KIF4A*,* KIF20A*, and *KIF11* were custom-designed, synthesized, and procured from Genomeditech (Shanghai, China). As a control group, we employed negative siRNA (si-NC) treatment as the mock control. Transfection was carried out using lipofectamine-3000 (Invitrogen Life Technologies, Carlsbad, CA, USA) according to the manufacturer’s instructions.

### RNA isolation and quantitative real-time PCR (qRT-PCR)

After rinsing with a solution of phosphate-buffered saline (PBS), we isolated total RNA using Trizol reagent (Sigma-Aldrich, Waltham, MA, USA). The reverse transcription and PCR procedures were performed following the instructions provided by the manufacturer (Vazyme, Nanjing, China). We determined the concentration of RNA using a Nanodrop2000 instrument (Thermo Scientific, Hudson, NH, USA). The custom synthesis of mRNA primers was outsourced to GENEWIZ company (Shanghai, China). The primer sequences were as follows: *KIF4A* Forword (F): CTGCCAACAAGCGTCTCAAGG, Reverse (R): CCTTCCATTCCACGGCTCTGA; *KIF11* F: ATCTGGTCTCCATTCCAAACTG, R: GCTTTGAGCTGCCATCCTTA; *KIF20A* F: GGCCGTTCCTGCATGATTGT, R: TGTCTGCCTTAGCCCCTTTCT; *GAPDH* F: TGACTTCAACAGCGACACCCA, R: CACCCTGTTGCTGTAGCCAAA. To normalize gene expression levels, we used *GAPDH* as a housekeeping gene and calculated relative quantification using the 2^−ΔΔCt^ method.

### Cell-Counting-Kit 8 (CCK-8) assay

We employed the CCK-8 assay as per the manufacturer’s guidelines (Sigma) to evaluate the growth of LUAD cells. The H1299 and A549 cells were initially distributed in 96-well plates at a concentration of 2 × 10^3^ cells/well. Subsequently, they were subjected to consecutive time points (24 h, 48 h, 72 h, 96 h, and 120 h) by adding 10 µl of CCK-8 solution. Subsequently, we measured the proliferation rate of tumor cells using absorbance readings at a wavelength of 450 nm.

### Transwell invasion assay

The upper lumen of an uncoated substrate (BD Biosciences, San Jose, CA, USA) was used to identify migrating cells, while Transwell membranes were precoated with 55 µl Matrigel (BD Biosciences, San Jose, CA, USA). After a culture period ranging from 4 to 24 h, the cells adhered to the top surfaces of the chambers were delicately eliminated using cotton swabs, while the cells attached to the bottom surfaces underwent staining for a duration of 5 min. Subsequently, these cells were captured and quantified using a microscope.

### Wound healing assay

We utilized a wound healing assay to assess the migratory potential of cells. Briefly, these transfected cells were cultivated in 6-well plates with a cell density of 5 × 10^4^ cells per well. Once the cell monolayer reached approximately 90% confluence, it was gently scratched using a Scratch meter and subsequently replenished with fresh serum-free medium under identical conditions. The migration area was quantitatively analyzed using Cellomics (Thermo Scientific).

### Colony formation assay

The A549 and H1299 cells were distributed evenly in 6-well plates with a cell density ranging from 400 to 1000 cells per well, followed by culturing LUAD cells for a duration of 14 d. Crystal Violet Staining Solution (Sigma) was then used to stain the cells for a period of 10–20 min. The colony numbers were subsequently determined.

### Statistical analysis

The statistical analyses were carried out utilizing R software version 4.1.1, and a significance level of *P* < 0.05 was deemed as statistically significant. To evaluate the disparities in OS among patient subgroups, Kaplan-Meier analysis was employed. The survival curves were compared using a two-sided log-rank test with a significance threshold set at *P* < 0.05. To compare the levels of immune cell infiltration between the two groups, we employed the Mann-Whitney test. IC50 values were obtained by non-linear regression of dose-response logistic functions, using GraphPad Prism 6.01.

## Results

### Identification of DEG-*KIF* genes

**Fig. 2:**
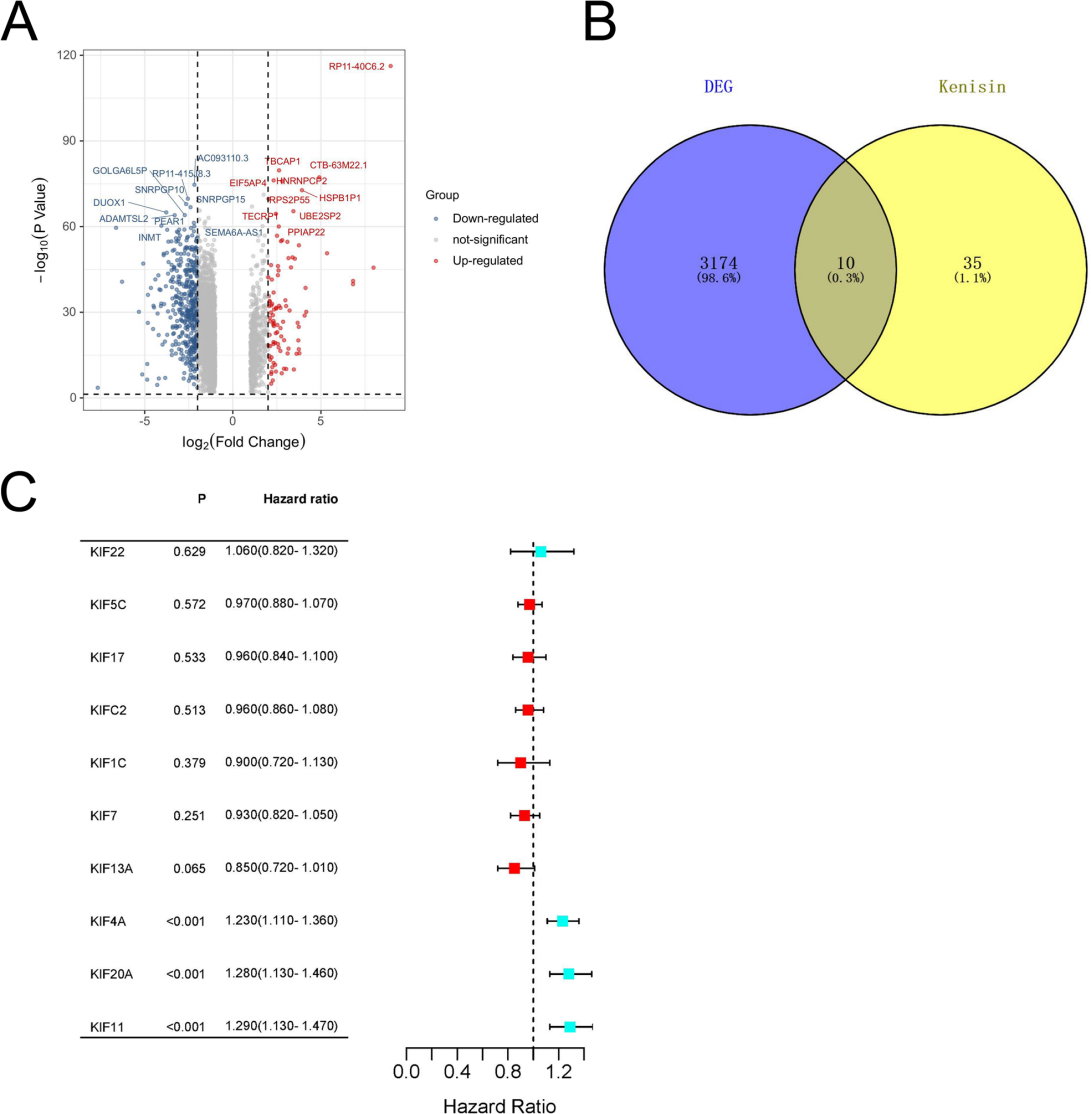
Identification of DEG-*KIF* genes. (**A**) The volcano plot is utilized to showcase the differential expression profile of genes in the LUAD dataset (cancer vs. normal); (**B**) The Venn diagrams’ shared kenisin were generated utilizing the web-based tool Venny 2.1.0; (**C**) Forest plot for the univariate Cox analysis of gene.

Data from two cohorts, TCGA-LUAD (*n* = 494) and GSE31210 (*n* = 226), were included in our study to analyze LUAD patients. Initially, we conducted a differential analysis using GEPIA2 on the TCGA-LUAD cohort, resulting in 3195 DEGs. The volcano map illustrating these DEGs is presented in Fig. 2A. Subsequently, we identified 10 common genes between the 3195 DEGs and a set of 45 *KIFs* (Fig. 2B). Finally, by performing univariate Cox analysis, we selected 3 *KIFs* that were associated with patient survival (Fig. 2C).

### Construction and validation of *KIFs* prognostics model

**Fig. 3:**
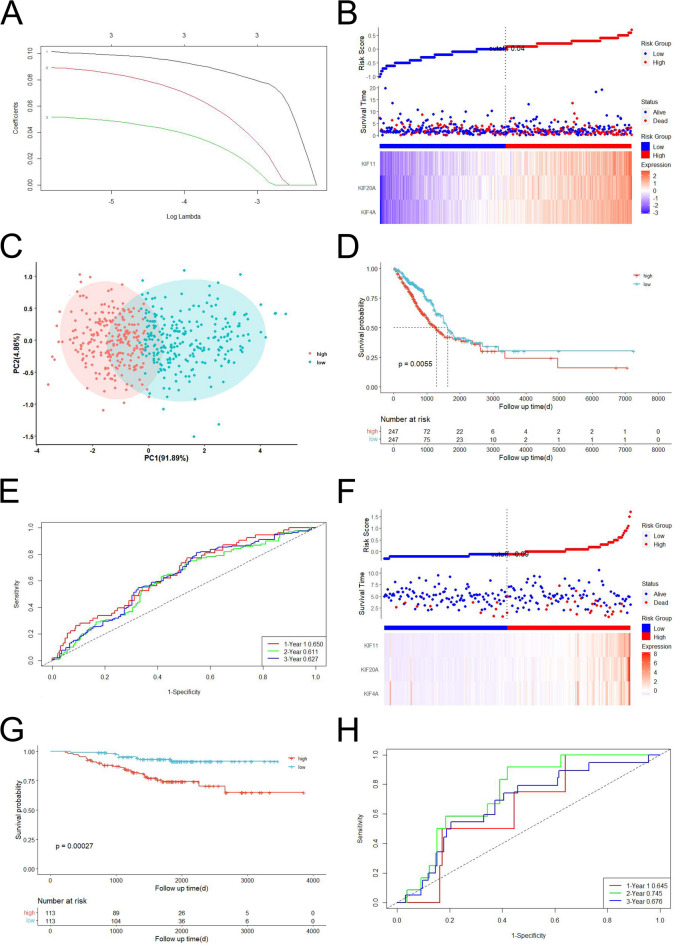
Construction and validation of gene signature. (**A**) LASSO regression of the 3 OS-associated genes; (**B**) Distribution of patients with LUAD based on their risk score, and the survival status for each patient (low-risk group: to the left of the blue line; high-risk group: to the right of the red line); (**C**) PCA plot for LUAD according to the risk score; (**D**) KM curves for OS of LUAD patients in the high- and low-risk groups; (**E**) ROC curves showed the predictive efficiency of this risk score; (**F**) Distribution of LUAD patients in the GSE31210 dataset based on the median risk score observed in the TCGA-LUAD cohort, along with their corresponding survival status (low-risk population: to the left of a blue line; high-risk population: to the right of a red line); (**G**) KM plot curves for comparison of the OS between low- and high-risk groups in GEO cohort; (**H**)Time-dependent ROC curves in GEO cohort.

We utilized lasso regression to develop a prognostic model consisting of 3genes (Fig. 3A). The risk score was then calculated using the following formula: (expression of *KIF4A*×0.101429576585459) + (expression of *KIF20A*×0.0892753671059616) + (expression of *KIF11* × 0.0515911609040658). According to the median risk score, a cohort of 494 LUAD patients was divided into two groups based on their level of risk (Fig. 3B). Our PCA effectively distinguished these patients into two distinct groups according to their risk level (Fig. 3C). The high-risk group exhibited significantly lower survival rates and higher mortality rates compared to the low-risk group (Fig. 3D).

Moreover, AUC values for predicting 1-year, 3-year, and 5-year survival were determined as 0.650, 0.611, and 0.627 respectively (Fig. 3E). 226 LUAD patients derived from a GEO cohort (GSE31210) were used as the validation dataset. According to the risk score distribution in GSE31210, 113 patients were assigned to the low-risk subgroup, while the remaining 113 patients were classified as high risk (Fig. 3F). Patients diagnosed with LUAD and belonging to the low-risk group demonstrated prolonged survival durations and reduced mortality rates compared to those in the high-risk group. Furthermore, Kaplan-Meier analysis indicated a significant discrepancy in survival rates between these two groups (*P* < 0.05; Fig. 3G). The performance of our model was effectively validated through ROC curve analysis, which exhibited favorable predictive accuracy (AUC = 0.645 for predicting one-year survival, 0.745 for three-year survival, and 0.676 for five-year survival) (Fig. 3H).

### Independent prognostic value of risk model and nomogram construction

**Fig. 4:**
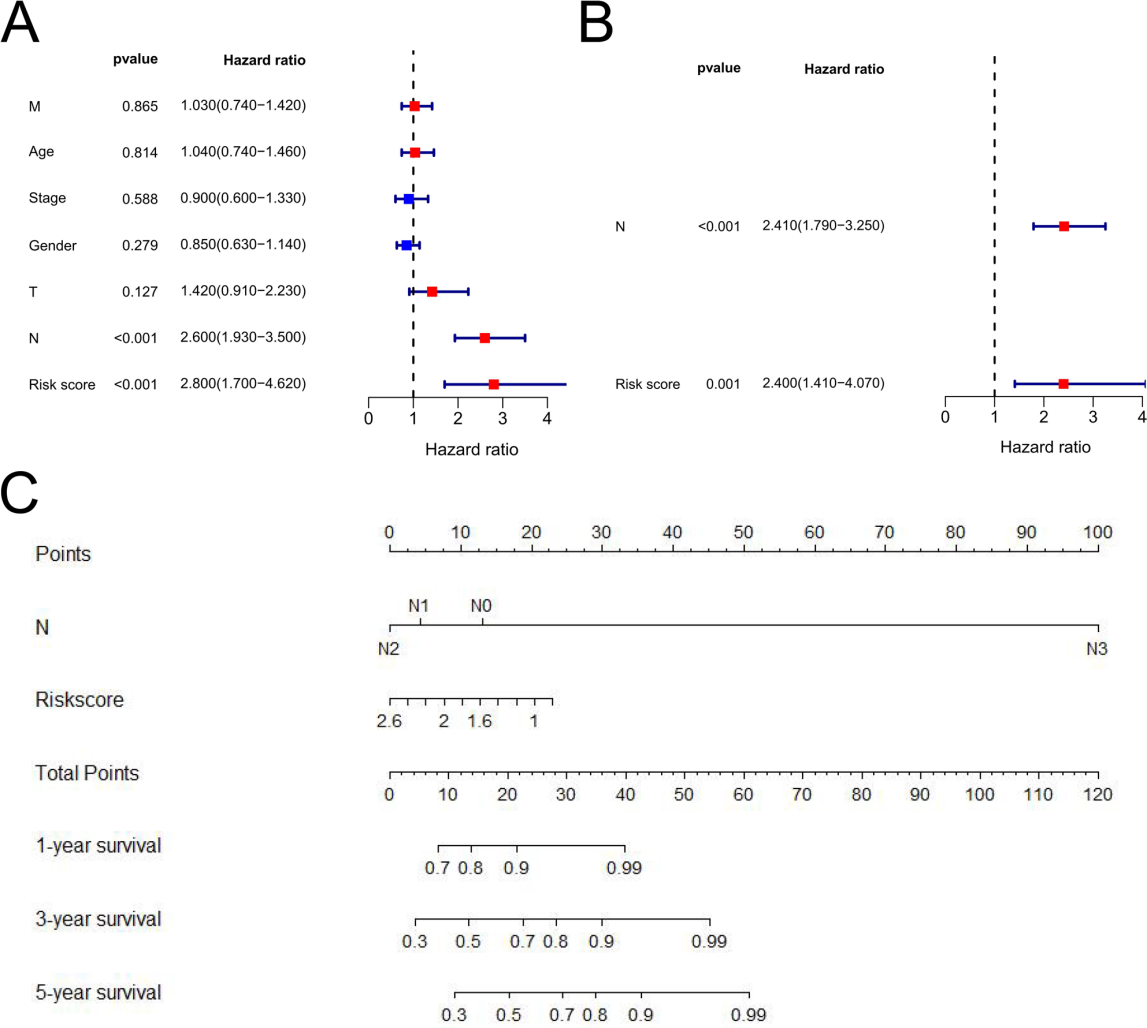
Independent prognostic value for gene signature. (**A**) Univariate analysis for the TCGA-LUAD dataset; (**B**) Multivariate Cox analysis for the TCGA_LUAD dataset; (**C**) Nomogram combining the gene signature with N.

To explore the potential of the gene signature model’s risk score as a standalone prognostic factor in LUAD, we performed univariate and multivariate Cox regression analyses. The findings from the univariate Cox regression analysis revealed that patients exhibiting high risk scores experienced reduced survival rates in the TCGA dataset for LUAD. Moreover, the multivariate Cox analysis revealed that even when accounting for various other influential variables, the risk score continued to exhibit substantial prognostic significance in patients with LUAD (Figs. 4A-B). To enhance the prognostic prediction for patients with LUAD, we devised a nomogram by integrating the risk score and an autonomous clinical risk factor (N) based on information extracted from the TCGA-LUAD dataset (Fig. 4C). Each variable identified through multivariate Cox analysis was incorporated into this nomogram. We then established two horizontal lines to assign specific points based on both the risk score and stage N. By summing up these points, we calculated total points for each patient. Finally, we generated projected survival probabilities at 1, 2, and 3 years by delineating a vertical line separating the cumulative point line from each prognostic line.

### Functional analyses according to the risk model

**Fig. 5:**
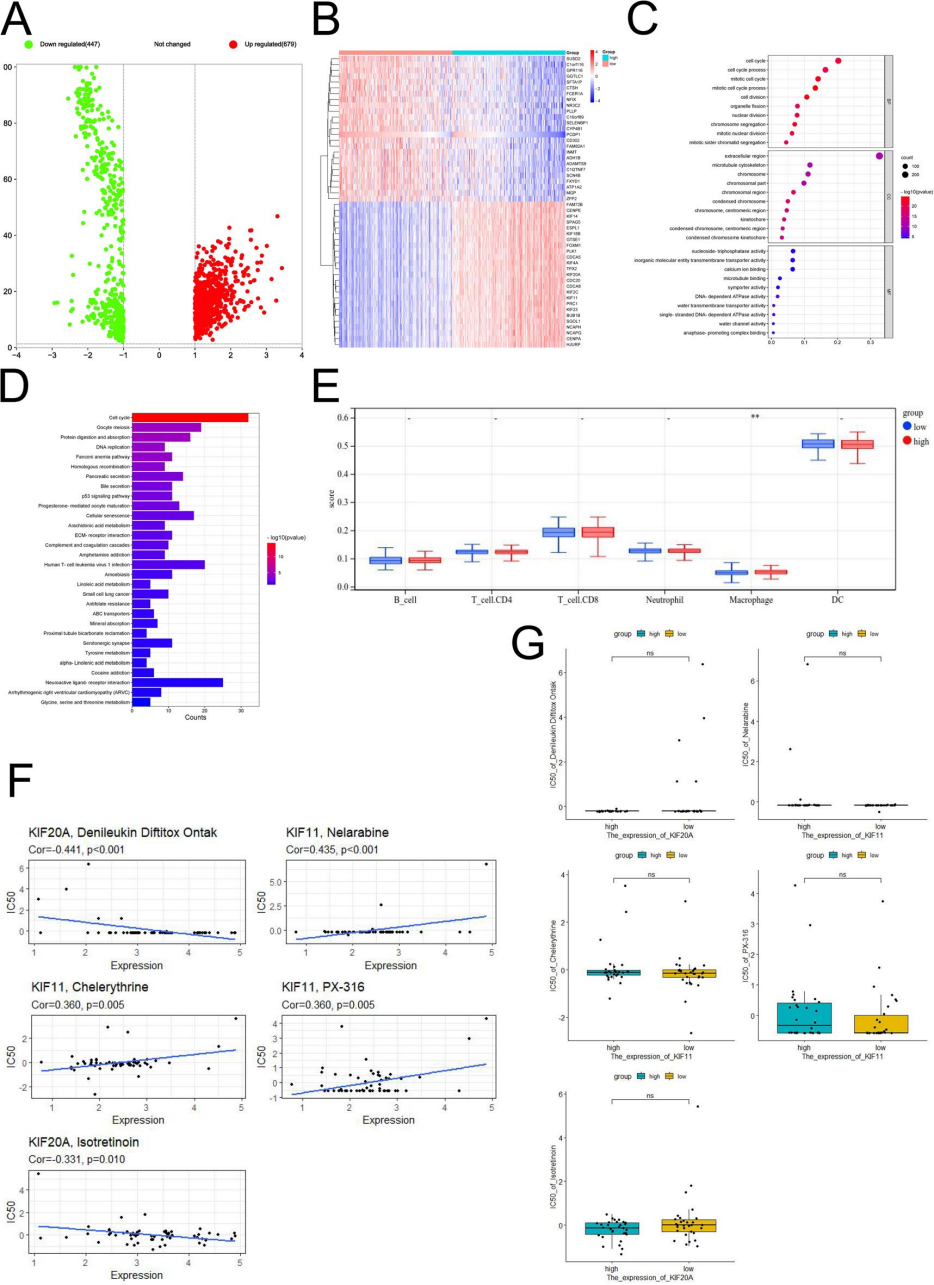
Bioinformatical analysis based on the DEGs. (**A**) The differential expression profile of genes in the different groups are presented as a volcano plot (high risk vs. Low risk); (**B**) Heatmap of the expression distribution of 50 DEGs and clinical features between the low-risk and high-risk groups; (**C**) Bubble graph for GO enrichment; (**D**) Barplot graph for KEGG pathways; (**E**) Differences of 6 immune cell subsets between high- and low-risk groups; (**F-G**) The association between kinesin expression and predicted FDA drug reaction.

To explore the differences in gene functions and pathways among distinct subgroups identified by the risk model, we employed the “limma” R package to detect genes with significant expression changes using a threshold of FDR < 0.05 and |log2FC|≥1. A total of 1850 DEGs were discovered between the low- and high-risk groups in the TCGA cohort (Fig. 5A). Figure 5B illustrated the expression levels of a selected set of 50 genes from both low and high-risk groups. Subsequently, GO enrichment analysis and KEGG pathway analysis were conducted using these DEGs. According to the results of the GO enrichment analysis, differentially expressed genes (DEGs) are highly enriched in the cell cycle and cell division pathways for biological function. Additionally, the KEGG pathway analysis also highlighted significant enrichment in the cell cycle pathway. Our analysis supported three kinesin genes (*KIF4A*, *KIF20A*, and *KIF11*) that may play a key role in tumor initiation and progression by regulating cell proliferation and division. The cellular component results indicate strong enrichment in the microtubule cytoskeleton pathway, suggesting that these three kinesin genes have significant impacts on tumor cell division, migration, and invasion. The outcomes revealed that these DEGs primarily correlated with neuroactive ligand-receptor interaction, ECM-receptor interaction, phagosome processes, etc. (Figs. 5C-D).

### Immunization and drug sensitivity analysis

According to the functional analyses conducted, we proceeded to compare the enrichment scores of 6 different types of immune cells in TCGA-LUAD cohorts. This was achieved by utilizing TIMMER. In the TCGA-LUAD dataset, it was noted that the low-risk group displayed a higher degree of immune cell infiltration, specifically macrophages, in comparison to the high-risk group (Fig. 5E). In order to explore the relationship between Riskscore and drug sensitivity, a thorough analysis was conducted. It was found that *KIF20A* expression displayed a positive association with drug response in patients who received Denileukin Diftitox Ontak, and Isotretinoin treatments. Conversely, *KIF20A* expression demonstrated a negative relationship with drug response in patients treated with Nelarabine, Chelerythrine, and PX-316. The relationship between the expression of kinesin and the expected response to medication is depicted in Figs. 5F-G. We treated lung adenocarcinoma cells with different drugs based on the IC50 values reported in the relevant literature^18–20^. The results showed that as *KIF20A* expression increased, the sensitivity to Denileukin Diftitox Ontak and Isotretinoin increased. Conversely, as *KIF11* expression increased, the sensitivity to Nelarabine, Chelerythrine, and PX-316 decreased (Supplementary Fig. 2A).

### High expression of *KIF4A*,* KIF20A* and *KIF11* in LUAD cells promoted proliferation of LUAD

**Fig. 6:**
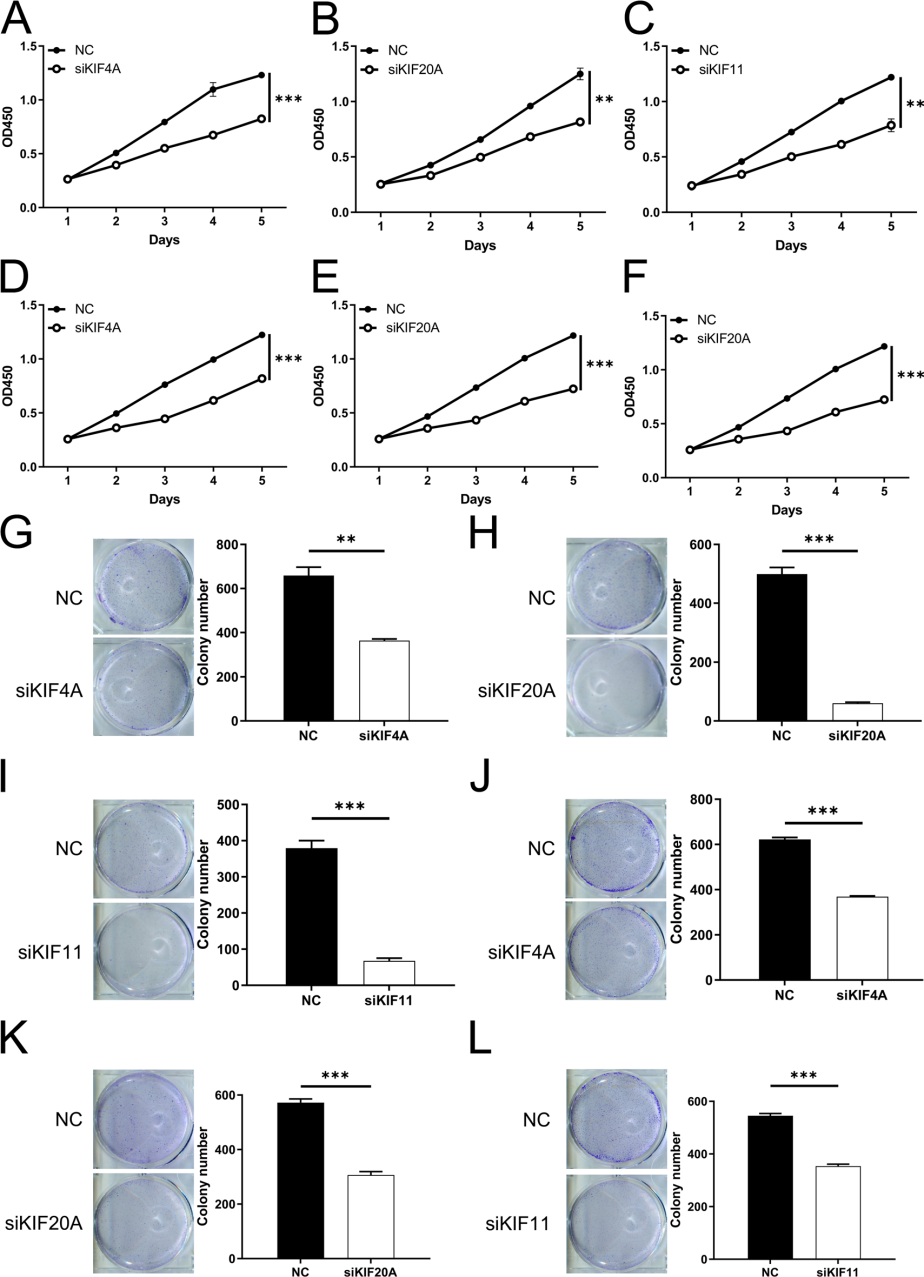
Down-regulation of *KIF4A*, *KIF20A*, and *KIF11* suppressed A549 and H1299 cells proliferation. (**A-F**) CCK-8 assay was utilized to determine the impact of knockdown of *KIF4A*, *KIF20A*, and *KIF11* on proliferation of LUAD cell line A549 (**A-C**) and H1299 (**D-F**) cells; (**G-L**) Colony formation assay was utilized to determine the impact of knockdown of *KIF4A*, *KIF20A*, and *KIF11* on proliferation of LUAD cell line A549 (**G-I**) and H1299 (**J-L**) cells. * *P* < 0.05, ** *P* < 0.01, *** *P* < 0.001.

To evaluate the levels of *KIF4A*,* KIF20A*, and *KIF11* expression in LUAD cell lines, we utilized two different LUAD cell lines (A549, H1299) along with a normal human bronchial epithelial cell line (BEAS-2B), employing the qRT-PCR assay. As shown in Supplementary Figs. 1 A-C, A549 and H1299 demonstrated increased mRNA levels of *KIF4A*,* KIF20A*, and *KIF11* compared to BEAS-2B. To further investigate the potential roles of *KIF4A*, *KIF20A*, and *KIF11* in LUAD cells, siRNAs were used to disrupt their expression in A549 and H1299 cells. We ultimately selected si-*KIF4A*-1, si-*KIF20A*-3, and si-*KIF11*-1 due to their superior interference efficiency (Supplementary Figs. 1D-E). In order to investigate the function of *KIF4A*,* KIF20A*, and *KIF11* in LUAD, we conducted cellular experiments. We utilized siRNA transfection to inhibit these proteins and then analyzed cell proliferation using both CCK-8 assay (Figs. 6A-F) and colony formation assay (Figs. 6G-L). Our findings indicate that inhibition of these factors can effectively reduce cell proliferation in LUAD.

### Ablation of *KIF4A*,* KIF20A*, and *KIF11* restrain LUAD cell migration and invasion

**Fig. 7:**
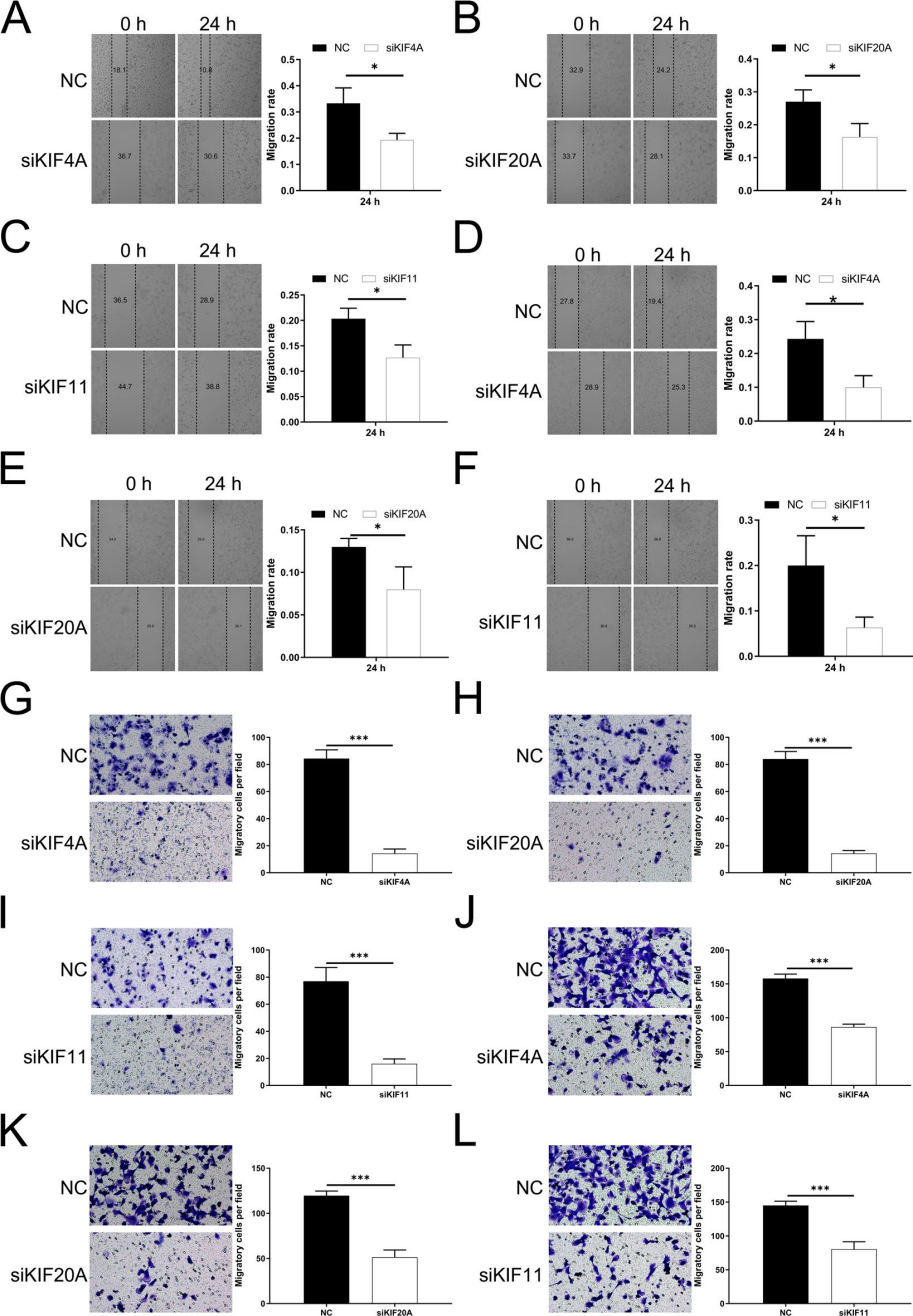
Down-regulation of *KIF4A*, *KIF20A*, and *KIF11* suppressed A549 and H1299 cells migration and invasion. (**A-F**) Wound healing assay was utilized to determine the impact of knockdown of *KIF4A*, *KIF20A*, and *KIF11* on migration of LUAD cell line A549 (**A-C**) and H1299 (**D-F**) cells; (**G-L**) Transwell assay was utilized to determine the impact of knockdown of *KIF4A*, *KIF20A*, and *KIF11* on invasion of LUAD cell line A549 (**G-I**) and H1299 (J-L) cells. * *P* < 0.05, ** *P* < 0.01, *** *P* < 0.001.

The migration of LUAD cells was evaluated using the wound healing assay to investigate the influence of *KIF4A*, *KIF20A*, and *KIF11* expression. Figures 7A-F displayed images captured at 0 h and 24 h, revealing that the si-*KIF4A*, si-*KIF20A*, and si-*KIF11* groups exhibited significantly reduced wound closure percentages compared to the control group after 24 h. Transwell invasion experiments were conducted to investigate the impact of reducing *KIF4A*,* KIF20A*, and *KIF11* on the motility of A549 and H1299 cells. The si-*KIF4A*, si-*KIF20A*, and si-*KIF11* groups exhibited a noticeable decrease in the number of invaded cells compared to the control group (Figs. 7G-L).

## Discussion

LUAD stands out as the prevailing histological subtype observed in cases of lung cancer. However, there is limited knowledge regarding effective biomarkers for LUAD. KIFs have been proven to play significant roles in tumorigenesis and disease progression. Pan et al. showed that a signature consisting of five *KIF* family members (*KIF4A*,* KIF26A*,* KIF1A*,* KIF13A*, and *KIF13B*) has the potential to be used as an indicator for predicting survival rates among individuals diagnosed with glioma^21^. Other findings suggest that targeting the protein known as *KIF14* could potentially offer novel therapeutic approaches for treating renal cell carcinoma (RCC)^22^. Song et al. also identified differential expression of 13 *KIF* genes in tumor tissues of breast cancer. Additionally, they observed associations between certain specific genes (*KIF15*,* KIF20A*,* KIF23*,* KIF2C* and *KIFA*) and prognostic factors related to breast cancer^23^. However, there is currently a lack of research examining the potential utilization of a prognostic signature based on these specific *KIFs* to predict prognosis in patients with LUAD. The objective of this study was to develop a *KIF*-related prognosis model in order to identify new biomarkers and investigate potential therapeutic targets.

In the present study, a total of 3195 DEGs were identified in the TCGA-LUAD dataset. We curated a list of 45 kinesin family members. Subsequently, a thorough investigation was carried out to detect and explore the intersection between these DEGs and *KIFs*, leading to the discovery of 10 common genes. Following this, univariate Cox regression analysis and LASSO regression were employed to select prognostically significant genes from this subset of DEG-*KIFs* for the development of an optimal risk model.

We utilized lasso regression analysis to construct a prognostic model involving 3 specific genes (*KIF4A*,* KIF20A*, and *KIF11*). The validity of this prognostic model was further confirmed in the GSE14520 dataset. Positioned at Xq13.1 on the genome, the *KIF4A* gene encodes a 140-kDa protein that predominantly localizes within the nucleus^24^. It plays crucial roles in various cellular processes such as proper segregation during mitotic cell division and regulation of chromosome condensation^25^. Previous research has provided evidence for the oncogenic role of *KIF4A* in various types of human cancers, including hepatocellular carcinoma, cervical carcinoma, breast cancer, oral cancer, and colorectal cancer^26^. In this study, we found that *KIF4A* could be used as a prognostic factor for LUAD, and its high expression would promote the proliferation and migration of cancer cells.

It has been observed that the activity of *KIF11* can impact the separation of centrosomes and the formation of the spindle^27^. Additionally, when *KIF11* is depleted, it can lead to abnormalities in cell division and arrest in the cell cycle, ultimately triggering cellular apoptosis. In a manner independent of mitosis, *KIF11* contributes to the regulation of axonal branching and growth cone motility, thereby impacting cellular migration^27^. Multiple research studies have consistently demonstrated a significant association between the expression of *KIF11* and prognosis in different types of cancer. The inhibitor known as K858 has demonstrated its ability to induce apoptosis and enhance chemo-resistance in head and neck squamous carcinoma cell^28^. Recent research findings indicate that suppressing *KIF11* significantly inhibits thyroid cancer cell proliferation while promoting apoptosis within these cells, highlighting the potential of targeting *KIF11* for effective thyroid cancer treatment^29^. Combining our findings with those reported above suggests that KIF11 plays an important role in the malignant progression and diagnosis of LUAD.

It has been demonstrated that abnormal expression of *KIF20A* is observed in various solid tumors, including glioma, hepatocarcinoma, breast cancer, nasopharyngeal cancer, gastric cancer, and lung cancer^30^. This aberrant expression is significantly associated with the prognosis of cancer patients^30^. Recent discoveries suggested that the promotion of fibrosarcoma progression, which is mediated by *KIF20A*, heavily relied on the critical involvement of the PI3K-Akt signaling pathway^31^. Consequently, targeting *KIF20A* could be a potential therapeutic strategy for fibrosarcoma treatment^31^. Some researchers also reported that autocrine activation of the androgen receptor induced by *KIF20A* contributed to the progression towards castration-resistant prostate cancer^32^. In summary, these studies collectively establish a clear link between tumor progression and three kinesin genes (*KIF4A*,* KIF20A*, and *KIF11*).

The emerging cancer stem cell theory, characterized by properties such as self-renewal and indefinite proliferation, poses significant challenges to the diagnosis and treatment of cancer^33^. Cancer stem cells (CSCs) contribute to resistance to cancer treatments, driving tumor recurrence, progression, and ultimately leading to metastasis and systemic disease^34^. CSCs participate in cancer immune surveillance and immune evasion through various mechanisms. Since CSCs have been identified and isolated in various carcinomas, including lung cancer, research on cancer stem cells offers valuable insights into addressing the drug resistance challenge in lung cancer^33^. Future research could explore the correlation between three kinesin genes (*KIF4A*,* KIF20A*,* and KIF11*) and cancer stem cells, potentially leading to new discovery.However, there were a few limitations in this study. Firstly, it is essential to obtain additional large-scale prospective studies from different sources to validate the findings since all data used in this study came from public datasets. Secondly, our research primarily relied on bioinformatics analysis and subsequent cell experiments were conducted to uncover the function of these cells. We confirmed that three kinesin genes (*KIF4A*,* KIF20A*,* and KIF11*) have some influence on the occurrence and progression of lung cancer. In future investigations, we plan to further explore their downstream regulatory mechanisms through cell experiments followed by validation using in vivo experiments.

## Conclusion

We developed a risk prediction signature comprising 3 *KIFs*, which exhibited high accuracy and consistency in forecasting the overall survival of patients with LUAD. Furthermore, our findings suggested that the under-expression of these 3 *KIFs* suppressed the proliferation, migration and invasion of LUAD cells. In conclusion, these results highlight the potential utility of this signature as both a biomarker and treatment target for individuals diagnosed with LUAD.

## Supplementary Information

Below is the link to the electronic supplementary material.

Supplementary Material 1

Supplementary Material 2

Supplementary Material 3

## Data Availability

The data that support the findings of this study are available in TCGA-LUAD (https://portal.gdc.cancer.gov/projects/TCGA-LUAD) and GEO (https://www.ncbi.nlm.nih.gov/geo/query/acc.cgi? acc=GSE31210).
