# Supplementary figures and images for: Kinesin genes KIF4A, KIF20A and KIF11 as prognostic biomarkers in lung adenocarcinoma by integrative bioinformatic analysis and experimental validation
Source: Sci Rep. 2025 Dec 29;15:44957. doi: 10.1038/s41598-025-29206-9 (PMC12748818; doi:10.1038/s41598-025-29206-9)

**A**

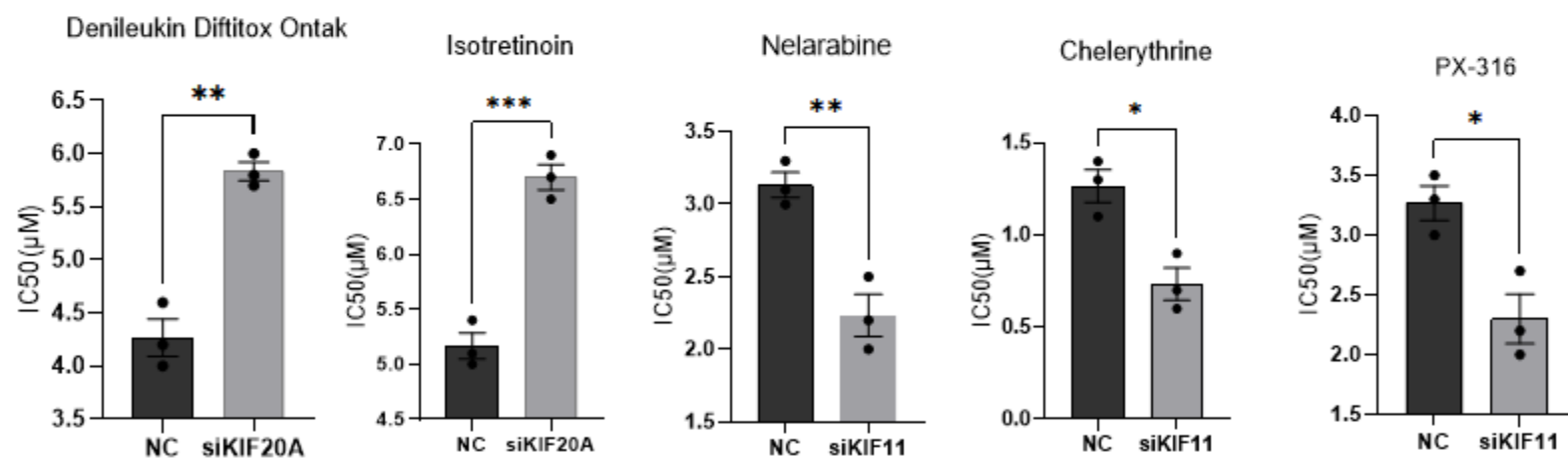

Supplement: Supplementary file 1 — Supplementary Material 1 [file 41598_2025_29206_MOESM1_ESM.pdf]

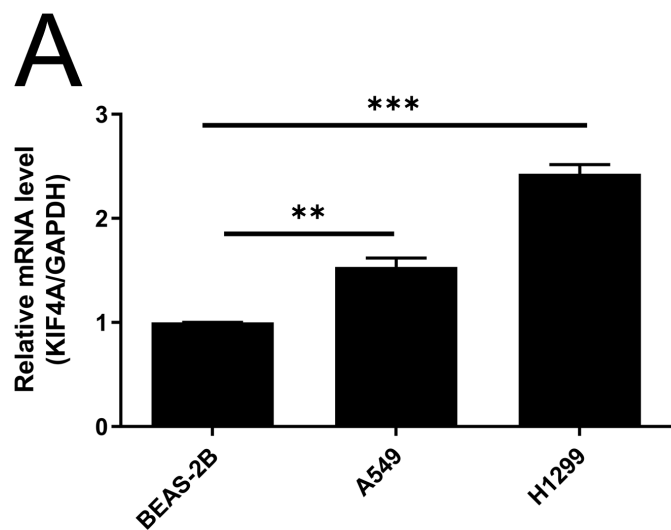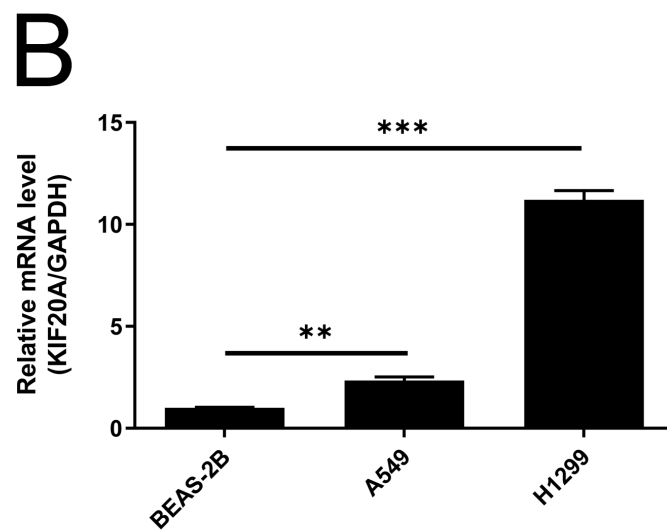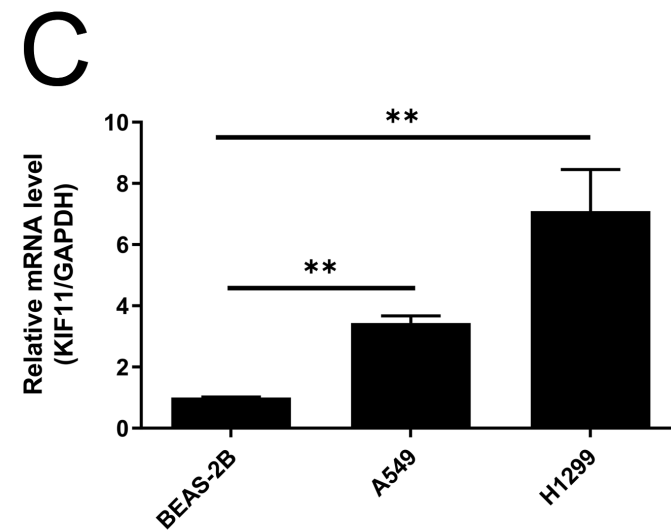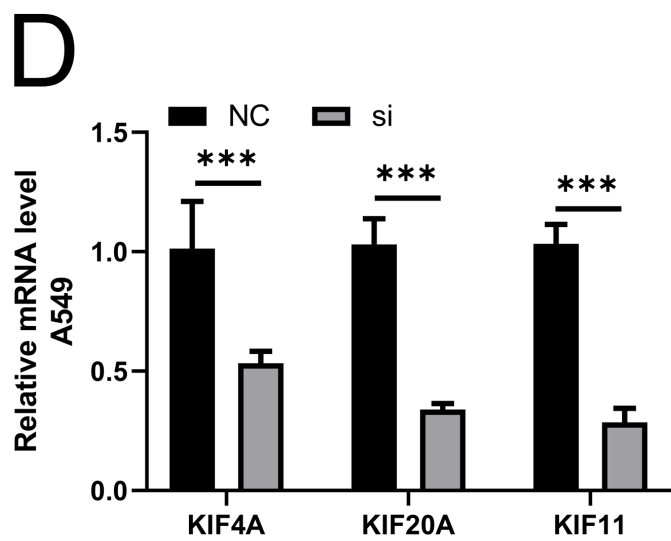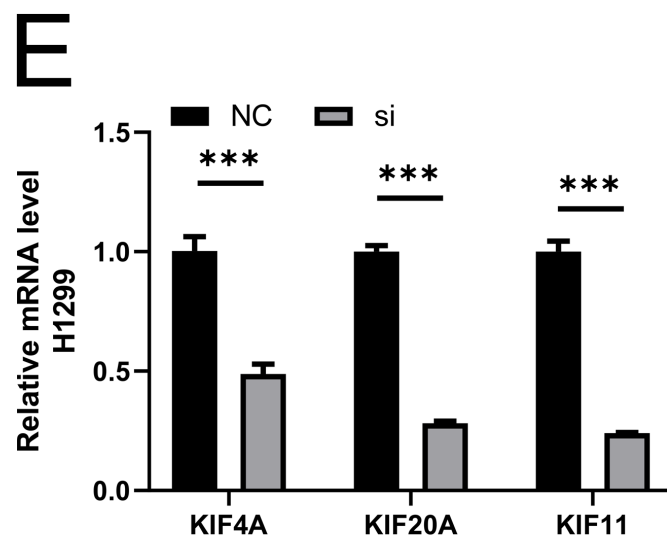

Supplement: Supplementary file 2 — Supplementary Material 2 [file 41598_2025_29206_MOESM2_ESM.pdf]
