# Supplementary material for: Kinesin genes KIF4A, KIF20A and KIF11 as prognostic biomarkers in lung adenocarcinoma by integrative bioinformatic analysis and experimental validation
Source: Sci Rep. 2025 Dec 29;15:44957. doi: 10.1038/s41598-025-29206-9 (PMC12748818; doi:10.1038/s41598-025-29206-9)
Supplement: Supplementary file 3 — Supplementary Material 3 [file 41598_2025_29206_MOESM3_ESM.pdf]

Supplementary Figure 1. High expression of *KIF4A*, *KIF20A* and *KIF11* in LUAD cells. (A-C) The expression levels of *KIF4A*, *KIF11* and *KIF20A* were analyzed using qRT-PCR in BEAS-2B, H1299, and A549 cell lines. (D-E) si-NC and si*KIF4A*, si*KIF20A*, si*KIF11* were transfected into H1299 cells, followed by qPCR analysis to determine the mRNA expression levels of *KIF4A*, *KIF11* and *KIF20A* in A549 and H1299 cells. * P <0.05, ** P <0.01, *** P <0.001.

Supplementary Figure 2. Detection of IC50 values after treating A549 cell lines with different drugs. (A) IC50 values were analyzed in A549 cell lines under different drug treatments. * P <0.05, ** P <0.01, *** P <0.001.
